# Phylogeny and taxonomy of two new *Plectosphaerella* (Plectosphaerellaceae, Glomerellales) species from China

**DOI:** 10.3897/mycokeys.57.36628

**Published:** 2019-08-07

**Authors:** Zhi-Yuan Zhang, Wan-Hao Chen, Xiao Zou, Yan-Feng Han, Jian-Zhong Huang, Zong-Qi Liang, Sunil K. Deshmukh

**Affiliations:** 1 Institute of Fungus Resources, Department of Ecology, College of Life Sciences, Guizhou University, Guiyang 550025, Guizhou, China Guizhou University Guiyang China; 2 Department of Microbiology, Guiyang College of Traditional Chinese Medicine, Guiyang 550025, Guizhou, China Guiyang College of Traditional Chinese Medicine Guiyang China; 3 Engineering Research Center of Industrial Microbiology, Ministry of Education, Fujian Normal University, Fuzhou 350108, Fujian, China Fujian Normal University Fujian China; 4 TERI-Deakin Nano Biotechnology Centre, The Energy and Resources Institute, Darbari Seth Block, IHC Complex, Lodhi Road 110003, New Delhi, India The Energy and Resources Institute New Delhi India

**Keywords:** Filamentous fungi, Plectosphaerellaceae, Multi-locus, Morphology, Taxonomy

## Abstract

The genus *Plectosphaerella* is the largest genus in the family Plectosphaerellaceae. Some species are plant pathogens, whereas others are soil-borne. Seven *Plectosphaerella* isolates were collected from various locations in the southwest of China. Using multi-locus phylogenetic (LSU, ITS, EF1α, RPB2) analyses combined with morphological characteristics, two new species, *Plectosphaerella guizhouensis***sp. nov.** and *Plectosphaerella nauculaspora***sp. nov.** are described, illustrated and compared with related species.

## Introduction

The genus *Plectosphaerella* Kleb., established in 1929, is the largest genus in the family Plectosphaerellaceae (Sordariomycetes, Glomerellales) (Giraldo and Crous 2019), consisting of some plant pathogen and soil-borne species. Previously, *Plectosphaerella* was proposed as a member of Hypocreaceae (Sordariomycetes, Hypocreales) (Gams and Gerlagh 1968, Barr 1990) or Sordariaceae (Sordariomycetes, Sordariales) (Uecker 1993). Zare et al. (2007) established the family Plectosphaerellaceae to accommodate *Acrostalagmus* Corda, *Gibellulopsis* Bat. & G. Maia, *Plectosphaerella* and *Verticillium* Nees. At that time, there were only five species in the genus *Plectosphaerella*, i.e. *P. cucumerina* (Lindf.) W. Gams, *P. cucumeris* Kleb., *P. himantia* (Pers.) Kirschst., *P. melaena* (Fr.) Kirschst. and *P. silenes* (Niessl) Kirschst. Carlucci et al. (2012) transferred all species of the anamorphic genus *Plectosporium* M.E. Palm, W. Gams & Nirenberg to *Plectosphaerella*. Subsequently, several new species and new combinations were introduced and transferred to the genus. To date, the genus *Plectosphaerella* contains 14 accepted species (Carlucci et al. 2012, Liu et al. 2013, Crous et al. 2015, Su et al. 2017, Wijayawardene et al. 2017, Giraldo and Crous 2019, Phookamsak et al. 2019).

Members of the genus *Plectosphaerella* are isolated from different habitats throughout the world, including plants, animals and soil. For example, *P. tabacinum* (J.F.H. Beyma) M.E. Palm, W. Gams & Nirenberg (the anamorph of *P. cucumerina*) has a cosmopolitan distribution with reports in Canada and the USA (North America), Belgium, England, Italy, The Netherlands and Switzerland (Europe), Egypt (Africa) etc. (Raimondo and Carlucci 2018, Giraldo and Crous 2019). It has been isolated from 11 species in 9 different plant genera: *Arabidopsis thaliana*, *Arabidopsis* sp., *Cucumis melo*, *Galium spurium*, *Hydrilla verticillate*, *Nicotiana tabacum*, *Pyrus malus*, *Solanum lycospersicon*, *Viola odorata*, *Viola tricolor*, *Austropotamobius pallipes* etc. (Alderman and Polglase 1985, Palm et al. 1995, Smith-Kopperl et al. 1999, Domsch et al. 2007, Giraldo and Crous 2019). Another common species, *P. plurivora* A.J.F. Phillips, Carlucci & M.L. Raimondo, has been reported from Australia, Belgium, Germany, Italy, The Netherlands, New Zealand, UK, the USA etc. and is isolated from soil, *Lolium perenne*, *Nicotiana tabacum*, *Solanum lycopersicum*, *Solanum tuberosum* etc. (Giraldo and Crous 2019). Raimondo and Carlucci (2018) reported that *Plectosphaerella* spp. could result in root and collar rot, plus vascular and leaf symptoms. Only two species, *P. oligotrophica* T.T. Liu, D.M. Hu & L. Cai and *P. humicola* Giraldo López & Crous, have been isolated from soils (Liu et al. 2013, Giraldo and Crous 2019).

During the investigation of keratinolytic fungi from different soils in China, seven isolates in the genus *Plectosphaerella* were obtained in Guizhou Province, China. The aim of our project was to identify these isolates, based on combined molecular phylogeny and morphological characteristics.

## Materials and methods

### Isolates and Morphology

Soil samples were collected from Qianlingshan Park (26°60'N, 106°69'E), Guiyang city and the affiliated hospital of Zunyi Medical University (27°70'N, 106°94'E), Zunyi city, Guizhou Province, China by Zhi-Yuan Zhang on 10 Sept. 2016. Samples were collected 3–10 cm below the soil surface and placed in Ziploc plastic bags. Isolation and purification of strains were undertaken according to methods described by Zhang et al. (2019). Sterile chicken feathers and human hairs were combined with the soil samples. Samples were placed in sterile Petri dishes, which were moistened with ddH_2_O. The baited soil sample Petri dishes were incubated at 25 °C for 1 month and remoistened as necessary. Two grams of sample were added to test tubes containing 9 ml of ddH_2_O. The mixture was then diluted to 1:10^4^ and 1 ml of suspension was evenly spread on plates containing Sabouraud’s dextrose agar (SDA, 10 g of peptone, 40 g of dextrose, 20 g of Agar, 1 litre of ddH_2_O) with anti-bacterial chloramphenicol and cycloheximide medium. Plates were incubated at 25 °C for 5 d. The axenic strains were then transferred to potato dextrose agar (PDA, Bio-way, China) plates for purification and to test-tube slants for storage at 4 °C.

Type collections of the novel species are deposited in the Mycological Herbarium of the Institute of Microbiology, Chinese Academy of Sciences, Beijing, China (**HMAS**). The ex-type living cultures and other strains of our study are deposited in the China General Microbiological Culture Collection Center (**CGMCC**) and the Institute of Fungus Resources, Guizhou University (**GZAC**). The axenic strains were incubated on PDA and Czapek agar (CA, Bio-way, China) at 25 °C in darkness. Macroscopic characterisation was undertaken after 14 d of incubation and the colony colours (surface and reverse) were observed. Preparations were mounted in ddH_2_O to study the mycelial morphology, conidiogenous cells, conidial structures and other microstructures from PDA cultures. Photomicrographs of diagnostic structures were made using an OLYMPUS BX53 microscope equipped with differential interference contrast (DIC) optics, an OLYMPUS DP73 high-definition colour camera and cellSens software v.1.18.

### DNA extraction, PCR amplification and Sequencing

Total genomic DNA was extracted from fresh fungal mycelia using the BioTeke Fungus Genomic DNA Extraction Kit (DP2032, BioTeke, China), following the manufacturer’s instructions. The internal transcribed spacer (ITS) regions and the 5’ end of the 28S nrRNA locus (LSU) were amplified and sequenced with the primer pairs ITS1/ITS4 (White et al. 1990) and LR0R/LR7 (Vilgalys and Hester 1990, Vilgalys and Sun 1994), respectively. Fragments of the translation elongation factor 1-alpha (EF1α) and the RNA polymerase Ⅱ (RPB2) genes were amplified with primer sets EF1-983F/EF-2218R (Rehner and Buckley 2005) and RPB2-5F/RPB2-7cR (Liu et al. 1999), respectively. Polymerase chain reaction (PCR) was performed in 25 μl reactions containing 1.0 μl DNA template, 1.0 μl of each forward and reverse primers (10 μmol/l), 12.5 μl 2× MasterMix (Aidlab Biotechnologies Co. Ltd., Beijing, China) and 8.5 μl ddH_2_O. Cycling conditions were as follows: initial denaturation at 94 °C for 5 min; followed by 35 cycles at 94 °C for 45 s, annealing depending on the locus (54 °C for ITS, LSU and EF1α, 56 °C for RPB2) for 45 s and extension at 72 °C for 60 s; and a final extension at 72 °C for 10 min. Sequencing was performed by TSINGKE Biological Technology (Kunming, China), using the corresponding primers.

### Phylogenetic Analyses

The DNA sequences, generated in this study, were assembled using Lasergene software (version 6.0, DNASTAR). Sequence data, mostly from Giraldo and Crous (2019), were downloaded from NCBI GenBank for molecular phylogenetic analyses (Table 1). Two sequences of *Brunneochlamydosporium nepalense* (isolates CBS 277.89 and CBS 971.72) were chosen as outgroup taxa. Sequences of each locus were aligned through MAFFT v.7.407 (Katoh and Standley 2013), using the default parameters and manually corrected in MEGA 6.06 (Tamura et al. 2013). The aligned sequences of multiple loci were concatenated by SequenceMatrix v.1.7.8 (Vaidya et al. 2011).

**Table 1.** Strains included in the phylogenetic analyses.

Species	Strain No.	GenBank Accession Number			
		LSU	ITS	EF1α	RPB2
*Brunneochlamydosporium nepalense*	CBS 277.89	LR025812	LR026683	LR026385	LR026111
	CBS 971.72 **T**	LR025813	LR026684	LR026386	LR026112
*Plectosphaerella alismatis*	CBS 113362 **T**	LR025932	LR026794	LR026489	LR026196
*P. citrullae*	CBS 131740	LR025933	LR026795	LR026490	–
	CBS 131741 **T**	LR025934	LR026796	LR026491	LR026197
*P. cucumerina*	CBS 137.33	LR025935	LR026797	LR026492	LR026198
	CBS 137.37 **T**	LR025936	LR026798	LR026493	LR026199
	CBS 139.60	LR025937	LR026799	LR026494	LR026200
	CBS 286.64	LR025938	LR026800	LR026495	LR026201
	CBS 355.36	LR025939	LR026801	LR026496	–
	CBS 367.73	LR025940	LR026802	LR026497	LR026202
	CBS 400.58	LR025941	LR026803	LR026498	LR026203
	CBS 567.78	LR025942	LR026804	LR026499	LR026204
	CBS 619.74	LR025943	LR026805	LR026500	LR026205
	CBS 632.94	LR025944	LR026806	LR026501	LR026206
	CBS 101014	LR025945	LR026807	LR026502	LR026207
	CBS 101958	LR025946	LR026808	LR026503	LR026208
	CBS 131739	LR025947	LR026809	LR026504	–
*P. delsorboi*	CBS 116708 **T**	LR025948	LR026810	LR026505	LR026209
***P. guizhouensis***	**CGMCC 3.19658 = GZUIFR-QL9.9.1 T**	**MK88043**1	**MK880441**	**MK930453**	**MK930460**
	**CGMCC 3.19659 = GZUIFR-QL9.9.2**	**MK880432**	**MK880442**	**MK930454**	**MK930461**
	**CGMCC 3.19660 = GZUIFR-QL9.9.3**	**MK880433**	**MK880443**	**MK930455**	**MK930462**
*P. humicola*	CBS 423.66 **T**	LR025949	LR026811	LR026506	LR026210
*P. melonis*	CBS 489.96 **T**	LR025950	LR026812	LR026507	–
	CBS 525.93	LR025951	LR026813	LR026508	–
*P. oligotrophica*	CBS 440.90	LR025952	LR026814	LR026509	LR026211
*P. oratosquillae*	NJM 0662 **T**	–	AB425974	–	–
	NJM 0665	–	AB425975	–	–
*P. pauciseptata*	CBS 131744	LR025953	LR026815	LR026510	–
	CBS 131745 **T**	LR025954	LR026816	LR026511	LR026212
*P. plurivora*	CBS 101.87	LR025955	LR026817	LR026512	–
	CBS 215.84	LR025956	LR026818	LR026513	–
	CBS 260.89	LR025957	LR026819	LR026514	LR026213
	CBS 261.89	LR025958	LR026820	LR026515	–
	CBS 291.38	LR025959	LR026821	LR026516	–
	CBS 292.66	LR025960	LR026822	LR026517	LR026214
	CBS 386.68	LR025961	LR026823	LR026518	LR026215
	CBS 406.85	LR025962	LR026824	LR026519	–
	CBS 417.81	LR025963	LR026825	LR026520	–
	CBS 642.63	LR025964	LR026826	LR026521	LR026216
	CBS 757.68	LR025965	LR026827	LR026522	LR026217
	CBS 101607	LR025966	LR026828	LR026523	LR026218
	CBS 131742 **T**	LR025967	LR026829	LR026524	LR026219
	CBS 131860	LR025968	LR026830	LR026525	LR026220
	CBS 143233 **T**	MG386133	MG386080	LR026526	LR026221
	**CGMCC 3.19654 = GZUIFR-H26.5.1**	**MK880436**	**MK880444**	**MK930456**	**MK93046**3
	**CGMCC 3.19655 = GZUIFR-H26.5.2**	**MK880437**	**MK880445**	**MK930457**	**MK930464**
	CBS 139623 **T**	KR476783	KR476750	LR026527	LR026222
	CBS 139624	MH878144	KR476751	LR026528	LR026223
*P. ramiseptata*	CBS 131743	LR025969	LR026831	LR026529	LR026224
	CBS 131861 **T**	LR025970	LR026832	LR026530	LR026225
*P. sinensis*	ACCC 39144	KX527892	KX527889	–	–
	ACCC 39145 **T**	KX527891	KX527888	–	–
***P. nauculaspora***	**CGMCC 3.19656 = GZUIFR-QL8.12.1 T**	**MK880424**	**MK880439**	**MK930451**	**MK930458**
	**CGMCC 3.19657 = GZUIFR-QL8.12.2**	**MK880425**	**MK880440**	**MK930452**	**MK930459**

**T**= type strain, strains and sequences generated in this study are shown in **bold**. **ACCC**: Agricultural Culture Collection of China, Beijing, China; **CBS**: Westerdijk Fungal Biodiversity Institute, Utrecht, The Netherlands; **CGMCC**: China General Microbiological Culture Collection Center; **GZAC**: Guizhou University, Institute of Fungus Resources; “–” represents the absence of GenBank accession.

Maximum likelihood (ML) analyses were constructed with IQ-TREE v. 1.6.11 (Nguyen et al. 2015). The best-fit model of substitution for each locus was estimated using IQ-TREE’s ModelFinder function (Kalyaanamoorthy et al. 2017) under the Bayesian Information Criterion (BIC). The selected models were TIMe+R2 for LSU, TNe+R2 for ITS, TIM2+F+R3 for EF1α and TN+I+G4 for RPB2. Bootstrap analyses was performed using the ultrafast bootstrap approximation (Minh et al. 2013) with 1,000 replicates and a bootstrap support (BS) ≥ 95% was considered as statistically significant.

For Bayesian Inference (BI), a Markov Chain Monte Carlo (MCMC) algorithm was used to generate phylogenetic trees with Bayesian probabilities using MrBayes v.3.2 (Ronquist et al. 2012) for the combined sequence datasets. The selection of the best-fit nucleotide substitution model for each locus was calculated by the Akaike Information Criterion (AIC) with Modeltest v.3.7 (Posada and Crandall 1988). The GTR+I+G model was selected for all datasets (LSU, ITS, EF1α, RPB2). Two runs were executed simultaneously for 5,000,000 generations and sampled every 500 generations. After the BI analyses, both runs were examined with Tracer v.1.5 (Drummond and Rambaut 2007) to determine burn-in and check for convergence. The final tree was submitted to TreeBASE, submission ID: 24412 (http://www.treebase.org).

## Results

### Phylogenetic analyses

Fifty-five strains (including the seven with new sequence data) were included in our multi-locus dataset (Table 1), which comprised 2536 positions, of which 322 were phylogenetically informative (35 of LSU, 54 ITS, 76 EF1α, and 157 RPB2). Tree topology of the Bayesian analyses was similar to that of the Maximum likelihood analyses.

**Figure 1.**
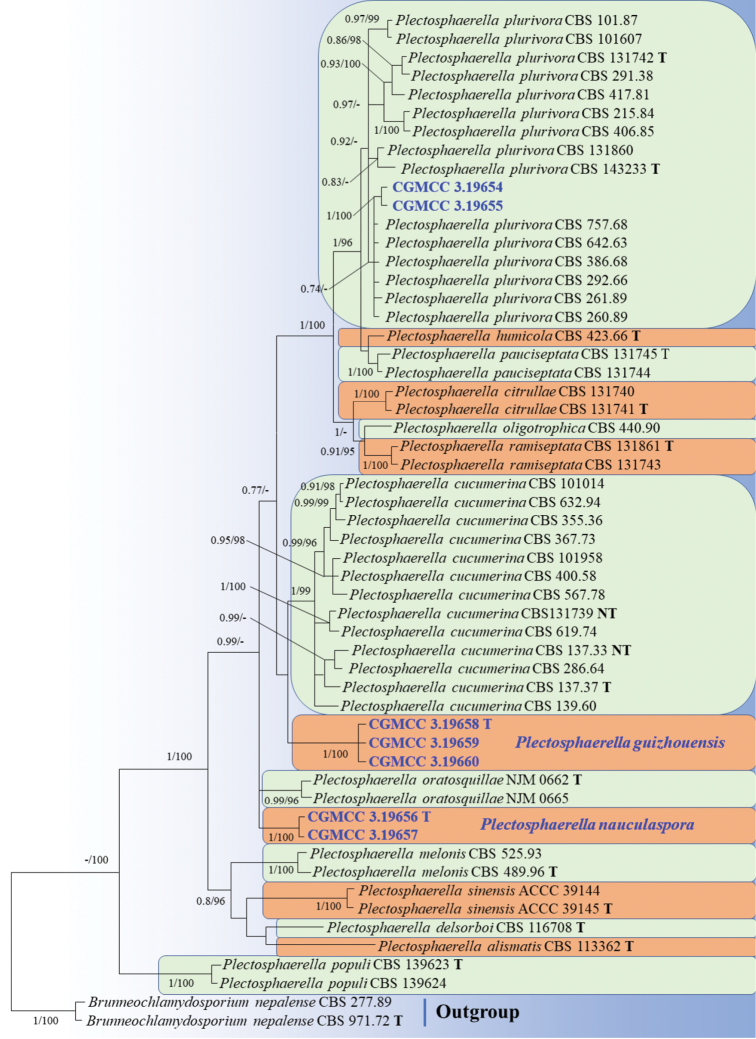
Phylogenetic tree of *Plectosphaerella* species derived from Bayesian analyses and Maximum Likelihood analyses, based on the combined sequences dataset of LSU+ITS+EF1α+RPB2. Bayesian posterior probabilities (BI pp) greater than 0.7 and Maximum Likelihood bootstrap support values (MLBS) greater than 95% are shown above branches. New isolates are in bold and blue. The tree used *Brunneochlamydosporium nepalense* (CBS 277.89 and CBS 971.72) as outgroup.

The analyses of concatenated dataset (Figure 1) showed that our isolates CGMCC 3.19658, CGMCC 3.19659 and CGMCC 3.19660 clustered in a single clade with maximum support (BI pp = posterior probability 1, MLBS 100). Similarly, the isolates CGMCC 3.19656 and CGMCC 3.19657 clustered in another single clade with high support (BI pp 1, MLBS 100). Furthermore, our isolates CGMCC 3.19654 and CGMCC 3.19655 clustered with other *Plectosphaerella plurivora* isolates from CBS in a single subclade supported by BI pp = 0.92.

## Taxonomy

### Plectosphaerella guizhouensis

Taxon classificationFungiGlomerellalesPlectosphaerellaceae

Zhi.Y. Zhang, Y.F. Han & Z.Q. Liang sp. nov.

943F1F407F8655ECB58DEAB76F3B3467

MB 830971

Figure 2

#### Etymology.

Referring to Guizhou, the province where the isolate was collected.

#### Description.

*Sexual morph* not observed. *Asexual morph* on CA. *Mycelium* hyaline, smooth, septate, branched and thin-walled, 1–2 μm (*x̄*=1.5 μm) wide. *Conidiophores* solitary, unbranched or rarely branched, hyaline, smooth, thin-walled, sometimes radiating out from hyphal coils. *Conidiogenous cells* growing from a short branch or directly from mycelia, phialides, discrete, polymorphic, cylindrical, sub-cylindrical or ampulliform; terminal or lateral, hyaline, smooth, solitary, straight at the apex, sometimes bent or helicoid, gradually tapering to the apex, 3.5–17 × 0.5–2 μm (*x̄* = 9.5 × 1.5 μm, n = 20), collarette cylindrical, 0.5–1 μm deep. *Conidia* aggregating in slimy heads, non-septate or 1-septate, fusiform or cylindrical, sometimes rounded at both ends, hyaline, smooth, thin-walled; 2–6.5 × 1.5–5 μm (*x̄* = 5.5 × 2 μm, n = 10) (1-septate), 3–5 × 1–1.5 μm (*x̄* = 4 × 1.5 μm, n = 10) (non-septate). *Chlamydospores* absent.

**Figure 2.**
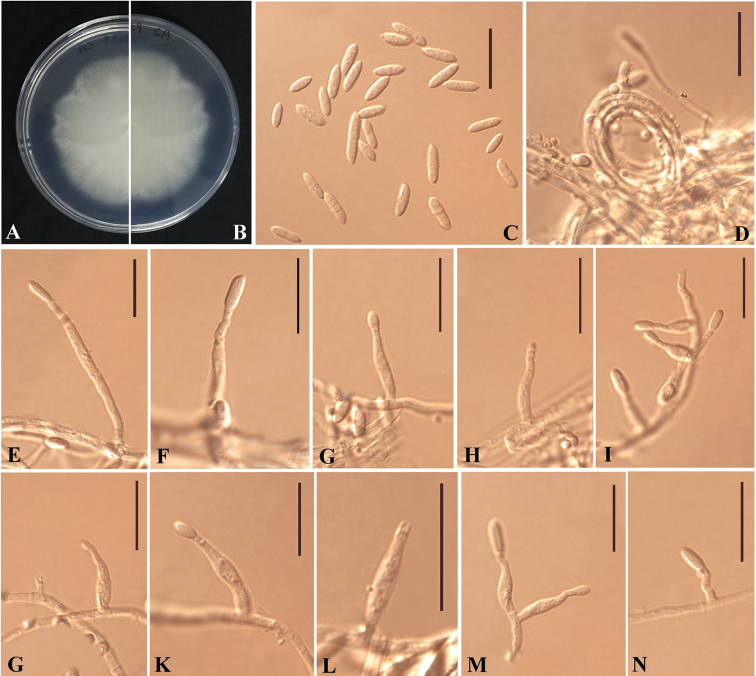
*Plectosphaerella guizhouensis* (HMAS 255618, holotype). **A–B** The front and reverse of colony on CA after 14 d at 25 °C **C** Septate and aseptate conidia **D** Hyphal coils **E–N** Phialides. Scale bars: 10 μm (**C–N**).

#### Culture characteristics.

Colonies on PDA reaching 74–75 mm diam. in 14 d at 25 °C, milk white, flat, aerial hyphae sparse, floccose at periphery, sub-rounded, margin regular, reverse milk white. Colonies on CA reaching 65–67 mm diam. in 14 d at 25 °C, white to milk white, flat, floccose, margin weakly undulate to faintly fimbriate, reverse milk white.

#### Typification.

CHINA, Guizhou, Guiyang, Qianlingshan Park, 26°60'N, 106°69'E, 1210 m a.s.l., on soil, 10 Sep. 2016, collected and isolated by Zhi-Yuan Zhang, HMAS 255618 (holotype), ex-type CGMCC 3.19658 (= GZUIFR-QL9.9.1); ex-isotype CGMCC 3.19659 (= GZUIFR-QL9.9.2) and CGMCC 3.19660 (= GZUIFR-QL9.9.3).

#### Notes.

Based on multi-locus phylogenetic analyses (Figure 1, see Results) and similar morphological characteristics, the three strains are regarded as the same species, which cluster together very well and form a single clade separated from other species of *Plectosphaerella* (Figure 1). Morphologically, *Plectosphaerella guizhouensis* differs from others species by the fusiform or cylindrical conidia, non-septate conidia (average 4 × 1.5 μm) and separate conidia (5.5 × 2 μm) (see Key). Therefore, based on combined phylogenetic and morphological evidence, *P. guizhouensis* is identified as a new species of *Plectosphaerella*.

### Plectosphaerella nauculaspora

Taxon classificationFungiGlomerellalesPlectosphaerellaceae

Zhi.Y. Zhang, Y.F. Han & Z.Q. Liang sp. nov.

74756F26C353509BA9FB530C2E9A533B

MB 830972

Figure 3

#### Etymology.

From “naucula”, referring to the navicular conidia.

#### Description.

*Sexual morph* not observed. *Asexual morph* on CA. *Mycelium* hyaline, smooth, septate, branched and thin-walled, 1–1.2 μm (*x̄*=1.5 μm) wide. *Conidiophores* solitary, unbranched or rarely branched, hyaline, smooth, thin-walled, hyphal coils not observed. *Conidiogenous cells* growing from short branch or directly from mycelia, phialides, discrete, polymorphic, cylindrical, sub-cylindrical or ampulliform; terminal or lateral, hyaline, smooth, gradually tapering to the apex, straight at the apex, sometimes bent or helicoid, 3–37 × 0.5–2 μm (*x̄* = 11 × 1 μm, n = 10), collarette minute, cylindrical, 0.5–1 μm deep. *Conidia* aggregating in slimy heads, 1- or 2–celled, mostly navicular, rarely fusiform or cylindrical, sometimes swollen at both ends, hyaline, smooth, thin-walled, 4–7 × 1–2 μm (*x̄* = 5 × 1.5 μm, n = 10) (1-septate), 3–5 × 1–1.5 μm (*x̄* = 4 × 1.5 μm, n = 6) (non-septate). *Chlamydospores* not observed.

**Figure 3.**
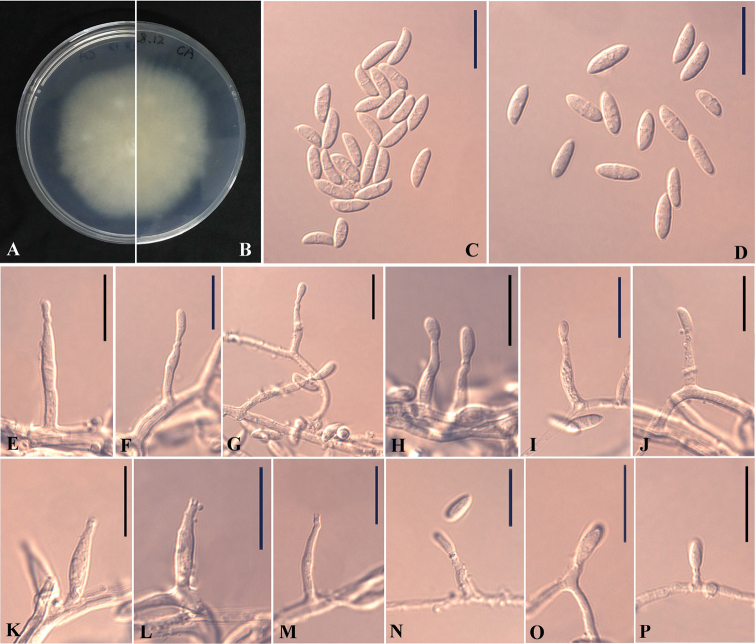
*Plectosphaerella nauculaspora* (HMAS 248154, holotype). **A–B** The front and reverse of colony on CA after 14 d at 25 °C **C–D** Conidia **E–P** Phialides. Scale bars: 10 μm (**C–P**).

#### Culture characteristics.

Colonies on PDA reaching 75–76 mm diam. in 14 d at 25 °C, milk white, flat, sub-rounded, margin regular, reverse milk white. Colonies on CA reaching 63–65 mm diam. in 14 d at 25 °C, milk white, aerial hyphae sparse, flat, margin weakly undulate to faintly fimbriate, reverse milk white.

#### Typification.

CHINA, Guizhou, Guiyang, Qianlingshan Park, 26°60'N, 106°69'E, 1220 m a.s.l., on soil, 10 Sep. 2016, collected and isolated by Zhi-Yuan Zhang, HMAS 248154 (holotype), ex-type CGMCC 3.19656 (= GZUIFR-QL8.12.1); ex-isotypes CGMCC 3.19657 (= GZUIFR-QL8.12.2).

#### Notes.

Phylogenetically, our two isolates CGMCC 3.19656 and CGMCC 3.19657 cluster together very well and form a single clade separated from the other species of *Plectosphaerella* (Figure 1). Morphologically, *Plectosphaerella nauculaspora* is the only species that produces navicular conidia in this genus. Therefore, based on both morphological and phylogenetic evidence, *P. nauculaspora* is proposed as a novel species.

## Discussion

In the present study, seven strains of *Plectosphaerella* fungi were isolated from soil in the Guizhou Province, China. Multi-locus phylogenetic analyses in combination with morphological data were used for identification. Our study resulted in the description of two new species, *P. guizhouensis* (3 isolates) and *P. nauculaspora* (2 isolates). In addition, our two isolates CGMCC 3.19654 and CGMCC 3.19655 closely clustered with *P. plurivora* and their morphological characters are similar to the original description *P. plurivora* (Carlucci et al. 2012).

*Plectosphaerella* spp. have diverse life styles and habitat sources – including pathogens of several plants, endophytes of plants, pathogens of animals (mainly involving *Austropotamobius pallipes* and *Oratosquilla oratoria*) and saprophytes on soil (Alderman and Polglase 1985, Palm et al. 1995, Domsch et al. 2007, Duc et al. 2009, Carlucci et al. 2012, Liu et al. 2013, Su et al. 2017, Liang et al. 2017, Raimondo and Carlucci 2018, Giraldo and Crous 2019). Although *Plectosphaerella* spp. were initially isolated from plants (from healthy or symptomatic tissue), subsequent studies found that they also widely distributed on soils and do not necessarily exhibit host specificity (Carlucci et al. 2012, Raimondo and Carlucci 2018, Giraldo and Crous 2019). However, *P. oratosquillae* can only be isolated from animals and it exhibits host specificity (Duc et al. 2009). Likewise, some species (mainly *P. oligotrophica* and *P. humicola*) have so far only been isolated from soils. In comparison with these previous studies, our two new species and one known species of *Plectosphaerella* were obtained from the soil beside a park road by the baiting technique (a method specifically designed for isolating keratinophilic fungi, Zhang et al. 2019). More studies are needed to assess whether our new species could be isolated from other habitats.

At present, more and more studies use combined data from morphological characteristics and molecular phylogeny for identifying new species (e.g. Carlucci et al. 2012, Liu et al. 2013, Su et al. 2017, Giraldo and Crous 2019, Phookamsak et al. 2019). Throughout the years, several loci have been used in the phylogenetic analyses of *Plectosphaerella* and its allies, containing ITS, LSU, EF1α, β-tubulin, CaM and RPB2 (Zare et al. 2007, Duc et al. 2009, Carlucci et al. 2012, Liu et al. 2013, Su et al. 2017). Giraldo and Crous (2019) revised the Plectosphaerellaceae and their results suggested that the phylogeny based on LSU+ITS+EF1α+RPB2 can be used for resolving intergeneric and interspecific relationships within the family Plectosphaerellaceae. As a result, we also used the LSU+ITS+EF1α+RPB2 dataset for phylogenetic analyses of *Plectosphaerella*.

### Key to the species of *Plectosphaerella*

**Table:** 

1	Growing on crustaceans	***P. oratosquillae***
–	On other substrates	**2**
2	Teleomorph known	**3**
–	Teleomorph unknown	**5**
3	Ascomata globose or subglobose to pyriform	**4**
–	Ascomata subglobose to ovoid, or obpyriform	***P. kunmingensis***
4	Asci 50–80 × 6–9 μm	***P. cucumerina***
–	Asci 31.4–43 × 6.2–8.2 μm	***P. plurivora***
5	Chlamydospores present	**6**
–	Chlamydospores absent	**8**
6	Conidia mostly septate	**7**
–	Conidia mostly aseptate	***P. melonis***
7	Conidia 13–19.5 × 2.5–3 μm	***P. alismatis***
–	Conidia 6–10 × 1.5–4 μm	***P. sinensis***
8	Phialides branched at tip	**9**
–	Phialides not branched at tip	**11**
9	Phialides 0–3-septate	***P. ramiseptata***
–	Phialides 0–1-septate	**10**
10	Oligotrophic, polyphialides infrequently seen, collarette 1–2.5 μm	***P. oligotrophica***
–	Non-oligotrophic, polyphialides frequently seen, collarette minute	***P. pauciseptata***
11	Conidia ellipsoidal	**12**
–	Conidia cylindrical, ellipsoidal, fusiform, navicular	**14**
12	Conidia mostly septate	***P. delsorboi***
–	Conidia aseptate	**13**
13	Conidia av. 4 × 2 μm	***P. populi***
–	Conidia av. 7.9 × 3.5 μm	***P. citrullae***
14	Conidia mostly navicular	***P. nauculaspora***
–	Conidia mostly cylindrical or fusiform	**15**
15	Septate conidia 2–6.5 × 1.5–5 μm, aseptate conidia 3–5 × 1–1.5 μm	***P. guizhouensis***
–	Septate conidia 7.5–11 × 2.5–3.5 μm, aseptate conidia 5–8 × 2.1–3.3 μm	***P. humicola***

## Supplementary Material

XML Treatment for Plectosphaerella guizhouensis

XML Treatment for Plectosphaerella nauculaspora
